# Obesity, Chronic Disease, and Economic Growth: A Case for “Big Picture” Prevention

**DOI:** 10.4061/2011/149158

**Published:** 2010-10-26

**Authors:** Garry Egger

**Affiliations:** ^1^Health and Human Sciences, Southern Cross University, Lismore, NSW 2480, Australia; ^2^Centre for Health Promotion and Research, P. O. Box 214, Balgowlah, Sydney, NSW 2094, Australia

## Abstract

The discovery of a form of chronic, low-grade systemic inflammation (“metaflammation”) linked with obesity, but also associated with several lifestyle-related behaviours not necessarily causing obesity, suggests a re-consideration of obesity as a direct cause of chronic disease and a search for the main drivers—or cause of causes. Factors contributing to this are considered here within an environmental context, leading to the conclusion that humans have an immune reaction to aspects of the modern techno-industrial environment, to which they have not fully adapted. It is suggested that economic growth—beyond a point—leads to increases in chronic diseases and climate change and that obesity is a signal of these problems. This is supported by data from Sweden over 200 years, as well as “natural” experiments in disrupted economies like Cuba and Nauru, which have shown a positive health effect with economic downturns. The effect is reflected both in human health and environmental problems such as climate change, thus pointing to the need for greater cross-disciplinary communication and a concept shift in thinking on prevention if economic growth is to continue to benefit human health and well-being.

## 1. Introduction

Obesity is currently pandemic, as are many of the chronic diseases often associated with this (e.g., type 2 diabetes) [1]. However, attributing the rise in chronic diseases to obesity does little to explain the true aetiology of the problem—the “cause of the causes” [2], which lies in more distal determining factors. This is indicated by recent findings that suggest a more complicated aetiological role for obesity than just a simple weight-disease association. The discovery of a form of low-grade systemic inflammation associated with obesity [3], as well as with other lifestyle and environmental factors (e.g., aspects of nutrition, inactivity, inadequate sleep, stress, depression, excessive alcohol intake, smoking, etc. [4, 5]) only some of which are linked to obesity, suggests that obesity may be just a marker of a type of environment and accompanying human lifestyle, which is mediated by aspects of the modern industrial environment to which humans have had little time to adapt. Furthermore, it has been shown, using the metaphor of inflammation, that this environment, is a driver not just of biological, but of ecological “disease,” manifest in excessive greenhouse gas emissions and potential climate change, as well as obesity and chronic disease [6].

In the current paper, which is proposed as a forum for a broader discussion in prevention, this argument is extended to consider the role of economic growth—beyond a point euphemistically defined as a “sweet spot” [7]—as a significant distal driver behind the development of high population levels of obesity and chronic disease, as well as potentiating factors for climate change. (It is important here to recognize the distinction between *economic growth, *which is the rate of increase in the market value of traded goods and services as measured by Gross Domestic Product (GDP), and which is widely used as the measure of progress throughout the world, and economic *development *(which can include desired improvements in renewable resources and nontangible assets such as literacy levels and access to heath care).) Although of undoubted benefit to human health and well-being in the early stages of development [8], economic growth, like all monetary investment, reaches a point, beyond which it begins to yield diminishing returns in relation to aspects of health and the environment. The current analysis outlines this and shows why. This is supported by empirical data, which define more clearly the transition stage [9] of developing economies such as that currently exists in China and India. The link between modern lifestyles, chronic disease, and environmental damage heralds the increasing need for a more dynamic discourse around the concept of growth, within which obesity and climate change have become “collateral damage” [7]. This now poses a threat to long-term prosperity, for which interdisciplinary solutions need to be urgently sought. In the meantime, “stealth interventions” [10], which are health interventions instigated for another purpose, but which have a positive side effect in health, might need to be considered to buffer the inevitable long-term changes required in the macroeconomic system. The use of personal carbon allowances [6, 11] is discussed as an example of an immediate and relatively painless option for doing this.

## 2. Obesity, Inflammation, and Chronic Disease

Obesity has been associated with ill-health since the days of Hippocrates who said that “.. *sudden death is more common in those who are naturally fat than in the lean*” [12]. Recent findings have modified this to associate different fat stores with disease, with particular emphasis on visceral adipose tissue or VAT [13] and more recently, intrahepatic fat [14]. Yet whilst this has increased predictive capacity, there are still reasons to suggest that obesity may be as much a marker of an aberrant lifestyle or toxic environment leading to disease, as a cause of disease in itself.

**Figure 1:**
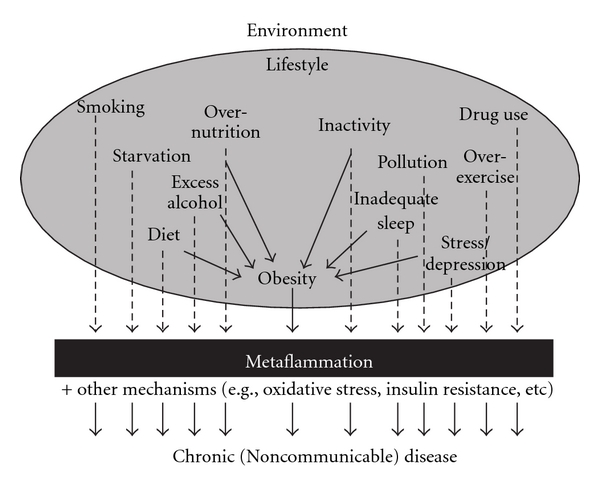
Environmental and lifestyle “inducers” of metaflammation, showing both independent and dependant effects through obesity (from [4]).

The discovery of a form of low-grade, systemic inflammation, since called “metaflammation” [15], associated with chronic disease outcomes, supports this. Originally thought to be a causal link between obesity and chronic disease [3], metaflammation has since been found to be associated with a wide range of (largely) lifestyle-related and environmental factors, characteristic of the modern industrial environment [6]. These are often, but not always, related to obesity, as shown by acute inflammatory reactions in the body before obesity has had time to develop (such as in the case of acute overnutrition or inactivity) or in some cases by the absence of obesity in the presence of these lifestyle behaviours (such as in the case of starvation, smoking, stress, inadequate sleep, excessive exercise). A list of factors associated with metaflammation, both in the presence and absence of obesity, is shown in Figure 1. Dotted lines in Figure 1 denote a connection between lifestyle or environmental stimulants and metaflammation/chronic disease without necessarily the mediation of obesity. Straight lines show a connection through obesity.

In contrast to the large (e.g., hundredfold) acute defensive reaction of classical inflammation, “metaflammation” is characterised by a small (~3-4-fold), but chronic and systemic rise in proinflammatory markers [15], many of which have now been identified that is from C-reactive protein (CRP) at the proximal level, to NFkB at the distal transcription level [16]. Anti-inflammatory markers indicate the opposing action of antagonising the metaflammatory process. These are less well known but adiponectin, leptin, and high-density lipoprotein (HDL) are amongst those that have been identified to date [17].

A table of factors causing pro- and anti-inflammatory responses based on research evidence is shown in Table 1.

From Table 1 it can be seen that a clear underlying distinction between pro- and anti-inflammatory stimuli is the length of time that humans, as a species, have engaged in these activities. Stimuli causing an immune inflammatory response on the left-hand side of the table for example are those with a relatively recent introduction into human lifestyles, whereas those causing an anti-inflammatory reaction on the right-hand side are those with which humans have evolved over many millennia. It should also be apparent that while some of those factors associated with inflammation may cause obesity, this has not been found to be a prerequisite for metaflammation, leading to a previous conclusion that it is the lifestyle factors causing obesity (overnutrition, inactivity), as well as other lifestyle factors associated with the modern environment (poor diet, excessive alcohol intake, inadequate sleep, stress, depression, passive and active smoking, air pollution, etc.) rather than obesity *per se*, which are the link with disease outcomes [4]. It is appropriate therefore to identify the driving factors behind these changes to shift the focus on chronic disease aetiology from obesity, to its more distal cause.

## 3. The Epidemiology of Chronic Disease

To cover the spectrum of causality, classical epidemiology typically involves investigating not just the cause of a disease, but the cause of that cause [2]. Based on the evidence presented above, this would lead to different levels of causality from proximal (diet, exercise, etc.) to medial (stress, anxiety, peer pressure, etc.) to distal causes (societal/economic influences, “modernity,” etc.), with vastly different implications for potentially successful population-level preventive interventions.

As stated above, the dichotomy between pro- and anti-inflammatory stimuli shown in Table 1 would suggest the time around the industrial revolution of the late 19th Century as a historical cutoff characterising the difference between these stimuli. This also defines the beginnings of the technoindustrial environment and the early stages of exponential growth of human populations. Hence industrialisation, and the economic and population growth accompanying this, should be considered as a distal cause of modern chronic disease. The fact that obesity in this scenario is a marker, as well as a cause of disease, suggests an interesting proposition that obesity is collateral damage from the modern environment, which in turn, is closely associated with human development, and more specifically, economic growth over the last ~200 years. Obesity and chronic disease, then result from our inability—or unwillingness—to adjust to the environment emanating from economic advancement, beyond a point. Metaflammation represents the body's response to this through a low level immune reaction to the lifestyle factors facilitated through such an environment.

## 4. Obesity, Chronic Disease, and Economics

There is ample evidence to support the notion that while economic development is generally positively associated with human health [8], this is not always the case, either over the short-term, in relation to booms and recessions [18–20], or over the long-term [21, 22]. It should be obvious, for example, that as the measure of economic growth, that is GDP, is determined by throughput, irrespective of outcome, it is possible that contributors to GDP could be health negative, or “illth” creating.(The term “illth” was first coined by the 19th Century English novelist John Ruskin (1857) who recognised that in the economic discussions of value of the time, no account had been taken of production of objects that cause harm or are socially undesirable. Ruskin referred to the outcome of those objects that are harmful or socially undesirable but still have value in exchange as “illth”.) Around 10% of GDP in developed countries, for example, is made up of revenue from tobacco, alcohol, and drug sales, many of which can have an adverse effect on human health (it is ironic that some cigarette manufacturers also manufacture surgical equipment, thus catering for both ends of the spectrum, making and doubling the benefit for GDP from smoking).

Of course, this has not always been the case, and hence to be comprehensive, any proposition of a link between economic growth and health needs to be dynamic. Growth has unarguably been positively related to human longevity and health overall, as witnessed by the big improvements in longevity in developed countries since the industrial revolution [8]. In developing economies however there is an “epidemiological transition” [23], where a switch occurs from a high prevalence of infectious disease to a growing prevalence of chronic diseases, after which mortality rates continue to decrease (although at a decreasing rate), but there is a growing increase in morbidity as reflected in the measure of years of life spent with a disability (YLD) component of disability adjusted life years (DALYs). The point leading up to this has euphemistically been referred to as a “sweet spot” in human development, where this reflects a point of optimal returns [7]. In economic terms this reflects the start of a point of diminishing marginal rates of return from continued investment in the growth paradigm.

The recent work by Tapia Granados and others [22, 24] has begun to objectify the point at which this might have already occurred in relation to some aspects of human health. In analysing Swedish mortality and economic data from 1800 to 2000, Tapia Granados and Ionides [24] have shown the beginnings of a diminishing rate of returns on the growth model in relation to mortality rates in the last half of the 20th Century. This coincides, not unexpectedly, with the levelling of improvements in health made from the decrease in infectious diseases associated with development, and the consequent increase in chronic diseases associated with modern lifestyles, driven as they are by the modern environment. It is this switch, from predominantly microbe-related infectious disease, to lifestyle-related chronic disease, and the consequent shift in the human immune reaction from an acute response to an invading organism, to a chronic reaction to nonmicrobial, lifestyle-related “inducers” [25] that differentiates the early from late stages of economic development. This has been shown in several developing societies and is currently being witnessed in China and India in accelerated form. 

Other support for the developing adverse effects of development, beyond a point, is gained from studying the situation in reverse. Cuba, for example, was forced into economic decline after Russia's withdrawal in 1989 resulting in a 1000 kcal/day average decrease in food intake. The following decade was characterised by an improvement in many aspects of chronic disease; overall mortality decreased by 20%, obesity was halved, and deaths from heart disease reduced by 35% and stroke by 18% [26]. Only cancers, which could be expected to have a longer lag-time in relation to causality, had not, at the time of study been affected. Nauru, a tiny island in the South Pacific, whose economy was based on superphosphate exported for fertilising the farm-lands of Australia and New Zealand, is another example. After exhaustion of its superphosphate supplies and consequent economic slump in the 1990s, Nauruans have decreased their obesity and diabetes levels dramatically from amongst the highest rates in the world [27]. Similarly, Hodge et al. [28] have shown the effects of development on obesity in villages in Papua-New Guinea. In quantifying measures of “modernity” such as cars, TV, microwaves, etc., Hodge et al. report a linear association between villages scoring high on modernity and high on measures of obesity, which is at least a marker, if not a determinant of chronic disease. In a closer examination of causality of such changes, researchers have found a link between lifestyles and disease. Using postwar Japan as an example, Tapia-Granados and colleagues found that times of economic prosperity correspond to increased consumption of tobacco, alcohol, and saturated fat; inactivity, work pressures, inadequate sleep, social isolation, traffic injuries, and social isolation [29], all of which have links with metaflammation and chronic disease [4].

**Figure 2:**
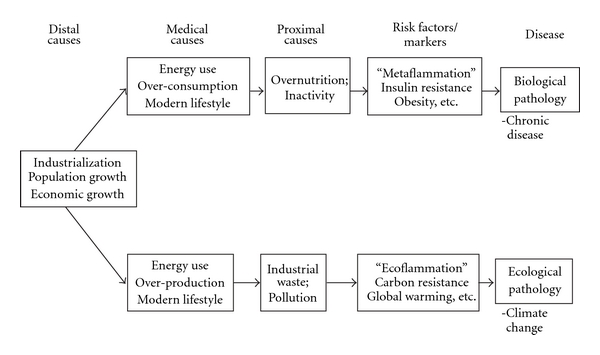
Relational epidemiology between chronic disease and climate change.

From a different perspective, negative developments in environmental degradation, such as manifest in climate change, also point to diminishing returns from unlimited growth. Studies on carbon and other greenhouse gas emissions, for example, show a highly positive association between atmospheric concentration of these and economic fluctuations [30], as might be expected. In a comparative analysis proposed recently [31], a converging causal hierarchy has been proposed between biological and ecological disruptions, which synthesises these effects and metaphorically ties them to “inflammation” (meta—versus ecoflammation), as shown in Figure 2.

An awareness of the convergence of health, climate, and macroeconomics gives rise to a need for consideration of all three phenomena.

## 5. Managing Diminishing Returns

It would be naive to propose the above argument as a reason to cease economic growth, or even revert immediately to a nongrowth, or steady-state economy. However, growing environmental, and health issues—as well as the often-unrecognised link between the two—make it important to place the issue on the prevention agenda. Consideration of the consequences of unfettered growth (both economic and population growth—which helps to drive this), which were raised in the 1970s [32], but then dismissed by religious, political, and economic arguments in the 1980s, need to be brought back to the fore within the context of human and environmental health and well-being. Discussions about climate change and environmental pollution, although currently based around technological growth as the potential solution, cannot be divorced from notions of exponential growth as a distal driver.

Immediate actions, which are within the realm of political possibility, such as carbon emissions policies, both corporate and personal, need to be expedited by health and environmental experts, without ignoring the long-term as well as short-term economic consequences. Corporate carbon policies are already on the statutes in many countries in one form or another and, if properly instituted, might be expected to have an impact not only on the environment, but on health, through the increased cost of energy-dense, and hence fattening, processed foods [6]. Yet corporate carbon emissions make up only 40%–50% of total emissions, the remainder being by individuals and households [33]. Hence the need for a personal carbon emissions policy aimed at reducing emissions from individuals and households. To this end, a personal carbon trading (PCT) scheme has been proposed by the Global Commons Institute in the UK [34] and embellished by others [11, 35]. PCT might be expected to increase personal energy expenditure (and hence reduce obesity by changing energy balance) by reducing fossil fuel usage. The system has been explained in more detail elsewhere [35], with a trial of such a scheme proposed in a “closed” environment of an island economy in the South Pacific [36]. This is designed to test the effect this should have on attitudes to over-consumption, which, together with the growth imperative, are the driving forces behind personal as well as environmental “illth.” 

## 6. Conclusion

In classic economic terms, body fat is an investment in the future. This yields healthy dividends (in this case, survival)—to a point, just as all good investments. But beyond this point, diminishing rates of returns begin to decrease the value of further investment. Body fat, to a level probably defined by individual genetics, is a necessary and healthy source of reserve energy. Recent findings suggest that while fat stays in the fat cells, for which they are designed, this is not a cause for concern. Only when lipid intolerant nonadipose organs are not protected against lipid “spillover” during sustained energy surplus does it become a problem [37]. In advanced economies, the bulk of such an energy surplus comes from the use of nonrenewable resources (fuel for transport and effort-saving devices, energy for production of energy-dense foods and drinks), the combustion of which also leads to greenhouses gas emissions. When these occur beyond a level of equilibrium with sequestration, or the ability of the earth's sinks to soak this up (the ecological “sweet spot”), they build up in the atmosphere, leading to ecological abnormalities such as severe weather events, species extinction, and climate change. The similarities between obesity and climate change, although metaphorical, are thus apparent. In seeking the driver of both, it is apparent that economic growth, beyond the point forewarned by the early economists such as J. S. Mill [38] as that where growth would need to be modified (as “nothing can grow forever”), is the ultimate distal cause. As summed up by one commentator: “*Growth beyond maturity is either obesity or cancer*” [39]. And while this is now becoming increasingly obvious amongst health scientists, it is imperative to involve other disciplines—economists, ecologists, politicians—in a discourse much broader than that which is traditionally considered as encompassing obesity and chronic disease prevention. Some initiatives are beginning in this direction (i.e., see [40]). However much more is likely to be required if human health and chronic disease are to be progressively improved.

## Figures and Tables

**Table 1:** Lifestyle and environmentally related metaflammatory “inducers.” (For a detailed list of references see references [10, 11], and at http://www.lifestylemedicine.net.au/health-information/lifestyle-medicine-evidence-base/inflammation-database/index.htm).

Proinflammatory	Anti-Inflammatory
A. Lifestyle	

Exercise too little (inactivity) too much Nutrition alcohol (excessive) excessive energy intake “fast food”/western style diet fat saturated trans fatty acids high fat diet high N6 : N3 ratio fibre (low intake) fructose glucose high glucose/GI foods glycaemic load glycaemic status meat (domesticated) salt sugar sweetened drinks starvation Obesity/Weight gain Smoking Sleep deprivation Stress/Anxiety/Depression/ “Burn out” “Unhealthy” lifestyle	Exercise/Physical Activity/Fitness “Healthy” obesity Intensive lifestyle change Nutrition alcohol capsaicin cocoa/chocolate (dark) dairy calcium eggs energy intake (restricted) fish/fish oils fibre (high intake) garlic grapes/raisons herbs and spices lean game meats low GI foods low N6 : N3 ratio Mediterranean diet fruits/vegetables mono-unsaturated fats nuts olive oil soy protein tea/green tea vinegar Smoking cessation Weight loss

B. Environment	

Age Air pollution indoor/outdoor Atmospheric CO_2_ Perceived organisational justice (low) “Sick building syndrome” Second hand smoke SE Status (low)	
